# Extension of Quality and Shelf Life of Tomatoes Using Chitosan Coating Incorporated with Cinnamon Oil

**DOI:** 10.3390/foods13071000

**Published:** 2024-03-25

**Authors:** Karthikeyan Venkatachalam, Somwang Lekjing, Paramee Noonim, Narin Charoenphun

**Affiliations:** 1 Faculty of Innovative Agriculture and Fishery Establishment Project, Prince of Songkla University, Surat Thani Campus, Makham Tia, Mueang, Surat Thani 84000, Thailand; karthikeyan.v@psu.ac.th (K.V.); somwang.s@psu.ac.th (S.L.); paramee.n@psu.ac.th (P.N.); 2 Faculty of Science and Arts, Burapha University Chanthaburi Campus, Khamong, Thamai, Chanthaburi 22170, Thailand

**Keywords:** tomato, edible coating, chitosan, cinnamon oil, storage, quality assessments

## Abstract

This study examined the effects of 2% chitosan (CS) coatings incorporated with varying concentrations of cinnamon oil (CO) (0%, 0.5%, 1.0%, and 1.5%) on the extension of the quality and shelf-life of tomatoes stored under ambient conditions. Control samples were untreated and coated with distilled water. All samples were stored for 14 days at 25 ± 1 °C, with quality assessments conducted every two days. The application of CS-CO treatments was notably effective in controlling weight loss (3.91–5.26%) and firmness loss (10.81–16.51 N), sustaining the color index score (11.98–16.78), and stabilizing the total soluble solids (4.64–4.71 brix), titratable acidity (0.374–0.383%), total phenolic content (75.89–81.54 mg/100 g), ascorbic acid concentration (21.64–33.69 mg/100 g), total antioxidant capacity (85.89–91.54%) and pigment levels, particularly chlorophyll (52.80–63.18 mg/100 g), compared to control samples (*p* < 0.05). Higher CO concentrations (1.0% and 1.5%) in the CS coating maintained a significant level of phytochemicals in the samples compared to the control group, while CS-CO at 0.5% performed similarly in preserving the other physicochemical qualities. Both CS and CS-CO treatments extended the shelf life of the tomatoes up to 14 days (<6.78 log10 CFU/mL), whereas control samples were only viable for storage for 6 days due to higher microbial growth (>7.8 log10 CFU/mL) (*p* < 0.05). Overall, CS-CO-treated tomatoes demonstrated superior quality preservation and shelf-life enhancement, with a notable improvement in overall qualities as compared to the CS and control samples.

## 1. Introduction

The tomato (*Solanum lycopersicum* Mill.) contains abundant levels of diverse phytochemicals, among which lycopene, polyphenols, and ascorbic acids are predominant [1]. It transcends its culinary role, offering significant medicinal benefits, especially in combating cancer and heart diseases [2]. Ripened tomatoes deliver the peak level of phytochemicals as compared to unripened ones [3]. Fully matured tomatoes have a shorter shelf life (5–7 days) due to various factors, including a high respiration rate, relative humidity, ethylene production, temperature fluctuations, micro-organism-induced degradation, and a high water content. The estimated post-harvest loss of tomatoes ranges between 20 and 65% of total production [4]. The limited post-harvest lifespan of these tomatoes poses a challenge for their distribution and sale, especially when they are best enjoyed at the breaker or turning stages of ripening. During these phases, the tomatoes exhibit their optimal qualities, including a firm texture, high sugar levels, and a well-rounded taste [5]. The tomatoes were often stored in refrigerated conditions to slow down the respiratory metabolism of tomatoes, or stored in an anaerobic environment. However, storing tomatoes and similar tropical/subtropical edible crops at temperatures below their critical threshold, specifically under 10 °C, leads to chilling injury (CI) [6]. This storage condition significantly impacts the tomato flavor even before visible symptoms appear. Furthermore, tomatoes stored at CI-inducing temperature could show a reduction in their ripe aroma and flavor and an increase in off-flavors compared to those stored at or above 20 °C [7]. Recent studies have focused on finding alternative treatments to refrigerated storage. Among these, the edible coating has garnered significant interest as a cost-effective and safer alternative [8]. Edible coatings are widely applied to extend the freshest produce at a variety of storage conditions, and, mainly, they are applied to extend the shelf life of fruits and vegetables [9]. Furthermore, when it is adequately prepared, it can be safely eaten as part of the product. Generally, an edible coating refers to a thin layer of natural polymeric materials, including proteins, polysaccharides, and lipids, which is hermetically applied to plant produce to provide protection.

Chitosan (CS), sourced from chitin crustacean shells, is a natural and edible polymer that is both non-toxic and eco-friendly [10]. CS is a linear polysaccharide produced through the deacetylation process of chitin. It is widely recognized as a versatile biopolymer, often employed as an edible coating for fruits and vegetables. This is due to its superior ability to form films and its biocompatibility, coupled with robust mechanical strength [11]. Furthermore, CS also exhibits various health-favorable functional properties, including antifungal, antibacterial, antitumor, antioxidative, and hypocholesterolemic activities [12]. Recent research has explored the enhancement of edible polymeric coatings by integrating natural additives, significantly boosting their protective capabilities [13]. CS has been the subject of extensive experimentation, particularly in its combination with various natural elements, such as other natural polymers, polyphenolics, nanoemulsions, and essential oils (EOs), to enhance its properties. Integrating EOs into edible coatings has significantly improved their antimicrobial effectiveness. Most components in EOs are volatile compounds, including alcohols, esters, aldehydes, monoterpenes, sesquiterpene hydrocarbons, and their oxygenated derivatives. The non-volatile component, constituting 5–10% of EOs, comprises coumarins, waxes, carotenoids, sterols, fatty acids, and flavonoids. The primary antibacterial substances in EOs can be categorized into three groups: terpenes (such as limonene and p-cymene), terpenoids (such as thymol and carvacrol), and phenylpropenes (such as eugenol and vanillin) [14]. The cinnamon EO (CO) stands out for its potent antimicrobial and antioxidant qualities. The primary source of CO’s antimicrobial action is its aldehyde content, particularly cinnamaldehyde content, b-caryophyllene, linalool, and various terpenes [15]. The antimicrobial impacts of CO and cinnamaldehyde have been well-documented in prior research. Isopentane, eugenol, eicosane, cinnamyl acetate, and anethole are further contributing to the potent antimicrobial activity of CO [16]. Additionally, incorporating this hydrophobic CO into polar-based coatings has enhanced their water barrier properties [17]. Studies have also demonstrated the efficacy of CS coatings enriched with CO in preserving the freshness and safety of various fruits and vegetables, including apples and blueberries [13,18]. The findings revealed that a CS coating combined with CO effectively inhibited bacteria, yeasts, and molds on fresh blueberry fruit, thereby significantly reducing the softening and decay of the berries. Furthermore, the antifungal properties of the CS-CO composite coating are remarkable, with antifungal activity increasing as the CO concentration rises [13]. Additionally, CS played a crucial role in preventing the volatilization and loss of CO in the composite coating, resulting in a synergistic antifungal effect. Notably, compared to CS alone, the CS-CO composite coatings were more effective in controlling post-harvest apple diseases, leading to a significant reduction in the diameter of apple spots caused by *Penicillium expansum* [18].

Therefore, the current study investigates the effectiveness of coating tomatoes with CS combined with CO at various concentrations (0%, 0.5%, 1.0%, and 1.5%). The main aim is to assess the efficacy of this treatment in extending the shelf-life, preserving fruit quality, and analyzing a spectrum of physicochemical and phytochemical parameters during storage under ambient conditions. The quality assessment included measurements of weight loss, color characteristics, firmness, pH, total soluble solids (TSS), titratable acidity (TA), TSS:TA ratio, chlorophyll content, lycopene content, β-carotene content, ascorbic acid (AsA) content, total phenolic content (TPC), total antioxidant capacity (TAC), and microbiological analysis.

## 2. Materials and Methods

### 2.1. Raw Materials, Chemicals, and Reagents

For raw material preparation, the tomatoes at approximately 80% maturity level were purchased from the fresh market in the southern part of Thailand. Tomatoes were carefully transported to the laboratory without any impact or abrasion injury, and, afterwards, they were sorted in uniform size, shape, and color. Tomatoes with any impact injury, insect, or pathogenic infections were removed. After selection, the tomatoes were thoroughly washed with distilled water, and then submerged in a sanitary solution made of 200 mg/L sodium hypochlorite for 10 min at ambient temperature. Then, the tomatoes were rinsed thoroughly with distilled water and left to dry at ambient temperature with the help of an electric fan for 15 min. Then, the tomatoes were collected and stored in the refrigerator until further use but utilized within the same day. For chemicals and reagents, the coating materials such as chitosan (CS), glycerol, tween 80 (polysorbate 80), and acetic acid were food-grade and purchased from Sigma Aldrich (Bangkok, Thailand). Sodium hypochlorite was procured from Merck (Bangkok, Thailand). Food-grade cinnamon EO (CO) was obtained from Merck (Bangkok, Thailand). Microbiological media (potato dextrose agar and plate count agar) and peptone water were purchased from HiMedia (Bombay, India). All the other chemicals and reagents used in this study were of analytical grade and procured from Sigma Aldrich (Bangkok, Thailand).

### 2.2. Preparation of Coating Solution: Application and Storage

The CS coating solution was prepared in accordance with the method of Xing et al. [18] with slight modification: 2% of CS (*w*/*v*) was added to the distilled water, followed by the addition of 1% acetic acid (*v*/*v*) and 1% glycerol (*v*/*v*), and then the mixture was thoroughly stirred in a steady phase using a magnetic stirrer at ambient conditions for 1 h to achieve a complete dispersion, and, once the dispersion level achieved, the CO at different concentrations (0%, 0.5%, 1.0%, and 1.5%) were separately added to the CS solution, followed by the addition of 0.2% (*v*/*v*) Tween 80. After that, the mixture was continuously stirred using the magnetic stirrer for another 30 min, followed by homogenizing at 22,000 rpm for 1 min using a tabletop homogenizer (IKA Works, Model T18, Bangkok, Thailand). Finally, the homogenate of CS-CO solutions was filtered using Whatman filter paper to avoid any air bubbles, and the final coating solution was left to stand at room temperature for 1 h before applying the tomatoes. For coating and storage, the tomatoes at refrigerated temperature were dipped in the CS-CO coating solutions in a ratio of 1:25 (fruit number/volume (mL) of coating solution) for 10 min, and then placed on the wire rack to drain for 2 min. After that, the samples were air-dried at 25 °C for 15 min using an air dryer cabinet. For control, distilled water was used instead of a coating solution, following the same coating duration and procedures as above. Once all the samples were coated and surface-dried, the tomatoes were weighed and labelled for replication and treatments, and stored (10 tomatoes/bag/replication) in perforated low-density polyethene bags. After that, all the samples were stored in ambient conditions (~25 ± 1 °C) for 14 days, and, at intervals of every 2-day, samples were measured for various quality determinations. The quality evaluation and storage period were terminated upon the appearance of any visible microbial growth on the surface.

### 2.3. Quality Determination

#### 2.3.1. Weight Loss

Weight loss was determined in tested samples by following the methodology described by Javanmardi and Kubota [19]. The weight loss during post-harvest storage was quantified by subtracting the current sample weights from their initial recorded weights. This loss was then expressed as a percentage. The percentage of weight loss was calculated using the following Equation (1), in which W_i_ refers to the initial weight of the tomato, and W_f_ is the weight after the storage period:

Weight loss (%) = [(W_i_ − W_f_)/W_i_] × 100 (1)

#### 2.3.2. Color Characteristics

Color determination of tomatoes was conducted in line with the methods described by Tadesse et al. [20]. During the storage phase, tomatoes were periodically subjected to color measurements encompassing L*, a*, and b* values. These measurements were taken using a colorimeter (HunterLab, Reston, VA, USA), which had been pre-calibrated with white and black reference plates. Color measurements were recorded for each tomato at four locations to ascertain the mean L*, a*, and b* values. Equations (2)–(4) were applied to calculate the hue angle, chroma, and color index, utilizing the obtained L*, a*, and b* values throughout the storage duration:

Color index = ((21.6a − 7.5b)/La) × 100(2)

Chroma = (a^2^ − b^2^) (3)

Hue angle = tan^−1^ (b/a)^2^ (4)

#### 2.3.3. Firmness

Fruit firmness was measured by following the protocol of Alenazi et al. [21]. A digital penetrometer (BKD020, Willow Bank Electronics Ltd., Napier, New Zealand), equipped with an 8 mm plunger, was used to measure the firmness of the tomatoes. The penetrometer was applied to the pared surfaces of the tomatoes, with the resultant firmness values expressed in Newtons (N).

#### 2.3.4. pH, Total Soluble Solids (TSS), and Titratable Acidity (TA), and TSS:TA Ratio

Juice from tomato samples were extracted using an electric juicer and then it filtered through muslin cloth and after that the filtrate was collected and used for measuring pH, TSS, and TA. For pH measurement, 100 mL of the tomato juice was collected and analyzed using a digital pH meter (Mettler-Toledo GmbH, Giessen, Germany). TSS was measured using a digital refractometer (PR-32a, ATAGO Co., Ltd., Tokyo, Japan) to determine the total soluble solids in the tomato juice, and the results were expressed in brix (°). For TA, approximately 95 mL of distilled water and a few drops of phenolphthalein indicator were added to a conical flask containing 5 mL of the prepared tomato juice. The TA of the tomato juice mixture in the flask was quantified by titration against 0.1 N sodium hydroxide (NaOH). TA values were calculated using the method described by Teka et al. [22] and expressed as a percentage of citric acid per mL of juice. The acid content of the fruit sample was calculated based on the volume of 0.1 N NaOH used for neutralizing the acid content in the sample and multiplying by a correction factor of 0.064 to estimate TA as percentage of citric acid. The TA was calculated using the following Equation (5):

(5) $$TA(\%)=\frac{mL\left(NaOH\right)\;\times \;N\left(NaOH\right)\;\times \;0.0064}{mL\;juice\;or\;g\;juice}\;\times \;100$$

where 1 mL 0.1 M NaOH is equivalent to 0.0064 g citric acid.

The TSS:TA ratio, which provides a comprehensive understanding of the balance between sweetness and acidity in the tomato samples, was determined by dividing the TSS values by the TA values, as described by Tigist et al. [23].

#### 2.3.5. Chlorophyll Content

Chlorophyll content in the tomato samples was quantified following the modified procedure of Ajdanian et al. [24]. Approximately 5 g of the tomato samples were homogenized using a homogenizer (IKA Works, Model T18, Bangkok, Thailand) in 20 mL of 99% methanol. The resultant mixture was then centrifuged at 6000 rpm for 10 min. The supernatant was collected for analysis, with absorbance measurements taken at specific wavelengths—663 nm for chlorophyll “a” and 653 nm for chlorophyll “b”—using a UV–Vis spectrophotometer (RF-15001, Shimadzu, Kyoto, Japan). The chlorophyll content was calculated using the following equations. The results were expressed in mg/100 g of fresh tomato samples (FW):

Chlorophyll a = 15.65*A*666 − 7.340*A*653 (6)

Chlorophyll b = 27.05*A*653 − 11.21*A*666 (7)

Total Chlorophyll = Chlorophyll a + Chlorophyll b(8)

#### 2.3.6. Lycopene Content and β-Carotene

Lycopene content and β-carotene in the tomato tissue were assessed by following Navarro et al. [25] with some modifications: 5 g of tomato sample tissues were homogenized using a homogenizer (IKA Works, Model T18, Bangkok, Thailand) with 25 mL of a solution containing acetone and hexane in a 4:6 ratio. The mixture underwent centrifugation at 5000 rpm for 10 min. Subsequently, the absorbance of the supernatant was measured at wavelengths of 663 nm, 645 nm, 505 nm, and 453 nm using a UV–Vis spectrophotometer (RF-15001, Shimadzu, Kyoto, Japan). The quantity of β-carotene and lycopene was calculated in mg/100 g of fresh weight of tomato samples (FW) based on these absorbance values, according to Equation (9), and, for the lycopene content, all the above measurements were the same, except the calculation, and the lycopene was used in Equation (10) for measurement:

Lycopene = 0.0458*A*663 − 0.204*A*645 − 0.372*A*505 + 0.0806*A*453 (9)

β-carotene = 0.216*A*663 − 1.22*A*645 − 0.304*A*505 + 0.452*A*453 (10)

#### 2.3.7. Ascorbic Acid (AsA) Content

AsA was quantified in tomato pulp using the method described by Chebrolu et al. [26]: 5 g of tomato pulp was homogenized using a homogenizer (IKA Works, Model T18, Bangkok, Thailand) with 5 mL of 1.0% hydrochloric acid, followed by centrifugation at 10,000 rpm for 10 min. The absorbance of the supernatant was then measured at a wavelength of 243 nm. A standard curve created using L-ascorbic acid (0.02, 0.04, 0.06, 0.08, 0.1, 0.2, and 0.5 mg/mL; R^2^ value was 0.997) as a reference was used to estimate the ascorbic acid content in the samples. The results were expressed as mg/100 g FW.

#### 2.3.8. Total Phenolic Content (TPC) and Total Antioxidant Activity (TAC)

##### Sample Extraction

Tomato tissue extraction was carried out in accordance with a method of Safari et al. [27] with some modifications. Initially, 5 g of tomato tissues were rapidly frozen using liquid nitrogen, then homogenized using a homogenizer (IKA Works, Model T18, Bangkok, Thailand) with 20 mL of 80% methanol (*v*/*v*). The homogenate was extracted in reduced light conditions on an orbital shaker for 1 h at 180 rpm. After shaking, the homogenate was filtered using Whatman no. 1 filter paper and centrifuged at 3000 rpm for 5 min. The resulting supernatant was collected for the subsequent measurement of TPC and TAC.

##### TPC

TPC in tomato extract was conducted using a modified Folin–Ciocalteu method proposed by Kaewseejan and Siriamornpus [28]. Initially, 150 μL of tomato extract supernatant was mixed with 750 μL of 10% Folin–Ciocalteu reagent, followed by 5 min of dark incubation. This was succeeded by the addition of 600 μL of 7.5% Na_2_CO_3_ and a subsequent 30 min of dark incubation at a stable environment of 26 ± 2 °C and 60 ± 5% RH. Absorbance readings at 765 nm were taken using a UV–Vis spectrophotometer (RF-15001, Shimadzu, Kyoto, Japan), and TPC values were calculated based on a gallic acid standard curve (0.01, 0.02, 0.05, 0.1, 0.2, 0.5, and 1.0 mg/mL; R^2^ value was 0.9951) and the results were expressed in mg gallic acid equivalence (GAE)/100 g FW.

##### TAC

Total antioxidant capacity (TAC) was assessed using the DPPH radical scavenging method adapted from Briones et al. [29]. Briefly, 1 mL of tomato extract supernatant was mixed with 1 mL of 1 mM DPPH solution in 80% methanol. After thorough vortexing, the mixture was incubated in the dark for 30 min at room temperature. Absorbance at 517 nm was measured using a UV–Vis spectrophotometer (RF-15001, Shimadzu, Kyoto, Japan). The reaction has replaced the sample with 80% methanol in the reaction mixture. TAC results were expressed as the percentage (%) of DPPH radical scavenging activity.

##### Microbiological Analysis

Microbiological shelf-life assessment of tomato samples was conducted following the method of Poubol et al. [30] with some modifications. A 10 g sample of tomato was mixed with 90 mL of 0.1% peptone water and homogenized using a homogenizer (IKA Works, Model T18, Bangkok, Thailand) for 60 s using a stomacher. Serial dilutions were prepared by transferring 1 mL of this mixture into 9 mL of 0.1% peptone water. Subsequently, 0.1 mL of each diluted sample was plated onto plate count agar and incubated at 7 °C for 10 days for aerobic psychrotrophic bacteria, and, for yeast and mold, potato dextrose agar was used and incubated at 25 °C for 5 days. Colony counts were then determined and expressed as log10 colony-forming units per mL (log10 CFU/mL).

### 2.4. Statistical Analysis

All data in this study are presented as mean ± standard deviation (SD) based on three replicates (*n* = 3). Statistical analysis was conducted using one-way analysis of variance (ANOVA). The least significant difference (LSD) test was employed for post hoc comparisons, with significance levels determined at *p* < 0.05. Statistical analyses were performed utilizing the SPSS software for Windows (Version 6, SPSS Inc., Chicago, IL, USA).

## 3. Results and Discussion

### 3.1. Weight Loss

**Figure 1:**
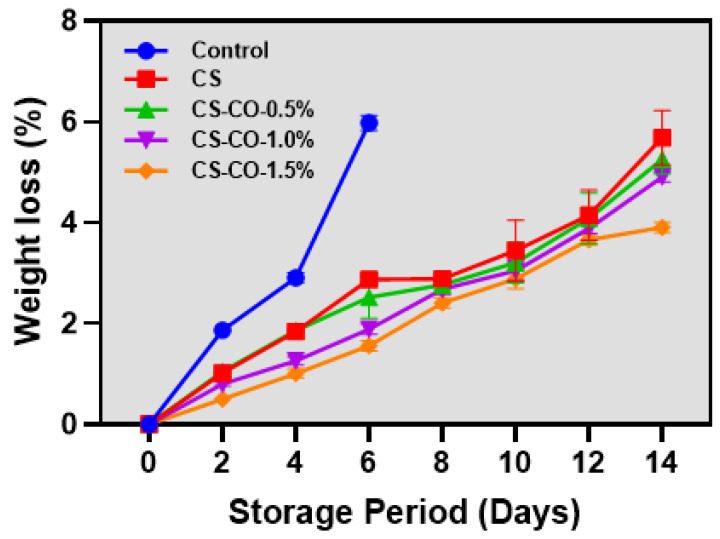
Changes in weight loss of the tomatoes treated with CS and CS-CO coating and stored under prolonged ambient conditions (~25 ± 1 °C). The results are presented as mean ± standard deviation (*n* = 3).

Changes in the weight of tomatoes coated with CS-CO coatings at varying concentrations are shown in Figure 1. Overall, this study found that the weight loss in tomatoes was significant and persistent throughout the storage period (*p* < 0.05). Generally, crop weight loss results from moisture loss, as crops continuously lose moisture due to respiration-induced transpiration processes [31]. The results showed that the control samples exhibited higher weight loss, reaching 5.98% on the sixth day of storage. In contrast, samples treated with CS and CS-CO significantly controlled the weight loss, maintaining it below 5.7% throughout the storage period. Among the CS-CO samples, those with a higher concentration of CO, particularly 1.5% in the CS coating, substantially controlled weight loss in tomatoes compared to other variants. Da-feng et al. [32] reported that, in comparison with the control samples, the CS-coated samples were able to control the respiration process by forming a barrier on the sample surface, thus preventing the exchange of O_2_ and CO_2_ between the coated layers of the sample and the environment and, thereby, extending the shelf life. This controls the respiration-induced transpiration levels, delaying crop dehydration and surface shriveling. Furthermore, adding CO to the coating emulsion further enhanced the protective effect against tomato weight loss. This improvement is attributed to the hydrophobic effect of CO, which enhanced the water barrier properties of the coating and also improved the stability of the coating material against degradation. Xing et al. [33] reported that CS-CO-treated jujube fruit samples significantly controlled the weight loss as compared to control groups by reducing the fruit metabolic-activity-induced transpiration rate. Singla et al. [34] tested the CS-CO coating on the pomegranate arils, and their finding showed a significant control against the fruit weight loss as compared to the control samples during storage.

### 3.2. Color Characteristics

**Figure 2:**
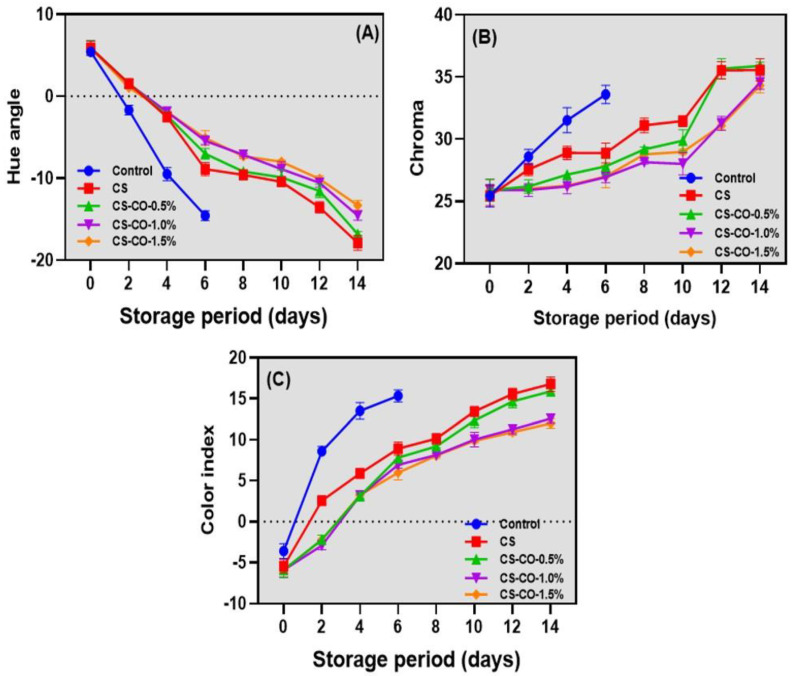
Changes in hue angle (**A**), chroma (**B**), and color index (**C**) of the tomatoes treated with CS and CS−CO coating and stored under prolonged ambient conditions (~25 ± 1 °C). The results are presented as mean ± standard deviation (*n* = 3).

The color profile plays a significant role in determining tomatoes’ ripeness, deterioration, and consumer acceptance [35]. The current study demonstrated that applying CS in conjunction with CO at various concentrations notably preserved the higher hue values in tomato samples compared to the control group. Despite variations, the trend in hue angle values consistently showed a decline across all samples; however, this decline was significantly more pronounced in the control group than in those treated with CS and CS-CO combinations. There were not many variations in the tomatoes’ hue values among the CS-CO samples. Viskelis et al. [36] reported that tomatoes stored under prolonged conditions at ambient storage might break the lycopene development, adversely affecting the hue angle. The changes in the chroma values of the CS-CO-coated tomato samples are shown in Figure 2B. Generally, an increase in chroma values in tomatoes represents an increase in or retention of redness values [37]. Overall, this study found that the extended storage period of tomatoes led to a continuous increase in their chroma values. The chroma values were slightly higher in the control samples compared to the treated samples. Among the CS samples, slightly higher chroma values were observed in the CS-CO-treated samples; however, the differences were insignificant. Ali et al. [38] reported that applying CS on crops can slow down the respiration rate and ethylene production, thereby delaying the ripening and senescence of plant produce. Consequently, this helps in controlling color development related to maturity. Furthermore, different concentrations of CO were slightly influenced by the change in chroma values in the tomatoes. Chrysargyris et al. [39] found that a high concentration of EO treatment initially delayed tomato chroma development by reducing lycopene levels, and this effect, prominently in the first week of storage, diminished over time with storage. Figure 2C illustrates the changes in the color index values of CS-CO-coated tomato samples during storage. Overall, the color index values of the tested samples continuously increased during the storage period. The control samples exhibited the highest values, followed by the CS and CS-CO samples. The CS-coated samples showed slightly higher values compared to the CS-CO samples. Specifically, 1% and 1.5% of samples treated with CS-CO effectively maintained lower color index values (*p* < 0.05). This implies that the chroma and color index of tomato fruits in samples treated with 0.5% CS-CO effectively increased over the storage duration, illustrating the ability of the tomato fruits to retain their redness at 0.5% compared with 1% and 1.5%. However, this indicates that 0.5% may not be sufficient for controlling and preserving the quality of tomatoes. As tomatoes ripen, lycopene accumulates and interacts with the internal membrane system, causing an increase in redness [20]. Several studies have reported that the color index of tomatoes is primarily associated with storage conditions, particularly temperature, as it is sensitive to ambient temperatures. Optimal plastid conversion in tomatoes occurs between 12 °C and 30 °C, indicating the critical role of temperature in influencing tomato coloration [37,40].

### 3.3. Firmness

**Figure 3:**
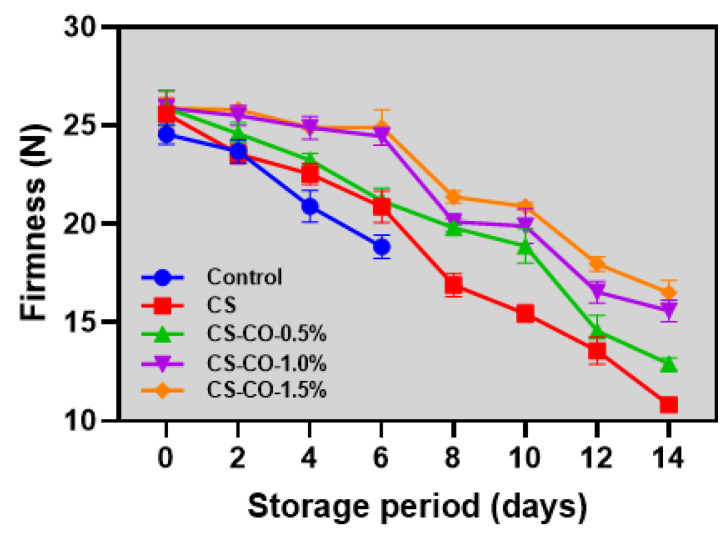
Changes in firmness of the tomatoes treated with CS and CS−CO coating and stored under prolonged ambient conditions (~25 ± 1 °C). The results are presented as mean ± standard deviation (*n* = 3).

Textural characteristics, particularly firmness, are an essential quality attribute and a deciding factor in the consumer acceptability of fresh produce [41]. The firmness changes in tomatoes treated with CS-CO at varying concentrations and stored under prolonged ambient conditions are shown in Figure 3. These results exhibited a continuous decline in firmness values throughout the storage period. At the initial storage period, the firmness values of the samples ranged between 24 and 25 N (*p* > 0.05). A continuous decline in tomato firmness was observed throughout the storage period, with firmness values between 10 and 17 N. Among the samples, the control samples exhibited the lowest firmness values on the sixth day of storage, followed by the CS- and CS-CO-treated samples. The CS-CO samples preserved tomato firmness more effectively than other samples, with an increased concentration of CO in the CS coating significantly enhancing fruit firmness. Ruelas-Chacon et al. [42] found that the firmness level of tomatoes continuously decreased during prolonged storage, regardless of whether they were coated or uncoated. This decline was slightly mitigated in the coated samples, as the coating could control the respiration-induced enzymatic activities, such as hydrolases, pectin esterase, and polygalacturonase, related to the ripening process. In coated tomatoes, the CS coating might act as a protective barrier, reducing oxygen permeation and, consequently, delaying respiration and fruit ripening [43,44]. Incorporating EO edible polymers improves their barrier characteristics and creates a beneficial microclimate on the treated samples’ surface, consequently decreasing the moisture loss and respiration rates and effectively inhibiting the increase in ethylene production in the treated samples. Choo et al. [45] observed that adding essential oils (EOs) to the chitosan/acetylated starch matrix likely improved protection against light, water vapor, and oxygen due to stronger bonding interactions. Moreover, several studies indicate that integrating CO into edible coatings can successfully diminish tissue softening and preserve firmness by inhibiting microbial growth, which is known to produce enzymes that degrade cell walls [46,47].

### 3.4. pH, TSS, TA, and TSS/TA Ratio

**Figure 4:**
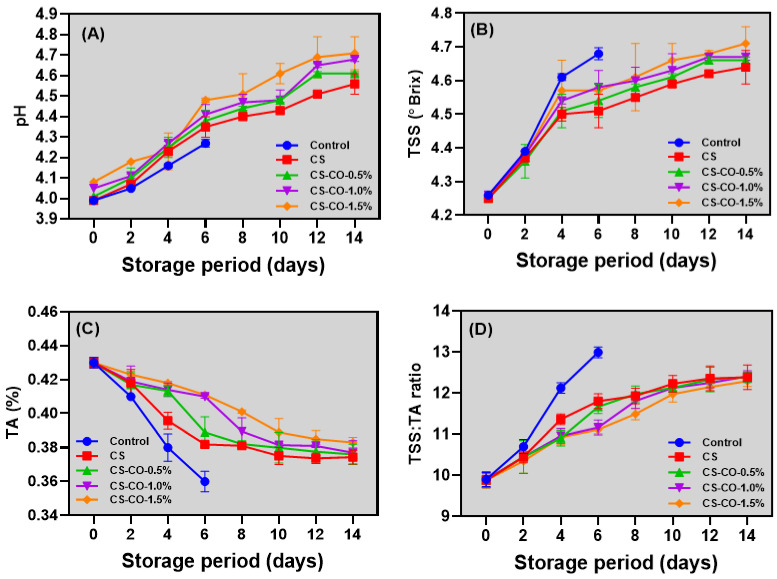
Changes in pH (**A**), TSS (**B**), and TA (**C**), and TSS:TA ratio (**D**) of the tomatoes treated with CS and CS−CO coating and stored under prolonged ambient conditions (~25 ± 1 °C). The results are presented as mean ± standard deviation (*n* = 3).

The changes in the pH, TSS, TA, and TSS/TA ratio values of the tomatoes coated with CS-CO at varying concentrations and stored under prolonged ambient conditions are shown in Figure 4. Overall, this study showed a significant increment in the pH values of the tomato samples during storage despite the differences in the tested variables (Figure 4A). Belay et al. [48] reported a rise in tomato pH during storage, possibly due to the effects of controlled O_2_ availability on the fruit’s respiration rate. Among the samples, the control group exhibited the lowest pH values compared to the other tested samples, and, between the CS and CS-CO samples, the CS-CO samples had the highest, followed by the CS-coated samples. Dovale-Rosabal et al. [49] reported similar observations, noting that under ambient conditions, the pH values in uncoated tomatoes were significantly lower than in coated ones. Among the different concentrations of CO-coated samples, the changes in pH values were very minimal. The observed pH variation in coated tomatoes can be attributed to intrinsic differences in the composition of the tested samples, which are influenced by cultural practices and environmental conditions. This variation aligns with the changes in TA observed during storage, suggesting a consistent relationship between pH evolution and acidity levels. The pH levels in tomatoes are directly associated with multiple factors, which, including storage duration, storage temperature, the conditions under which they ripen, and the severity of their respiration rates and changes in any of these conditions, can influence the acidity or alkalinity of tomatoes. For instance, longer storage times and higher temperatures can accelerate metabolic processes, leading to changes in pH [50,51]. Similarly, the environmental conditions, particularly temperature during ripening and the intensity of the tomatoes’ respiration rates, can alter the chemical composition, affecting the pH levels [52,53]. The titratable acidity decreased in accordance with the pH increases. A controlled effect of CS-CO on the pH level of tested tomatoes could be the protective barrier against the environment, including respiratory gases, particularly O_2_, and microbial growth [54]. The measurement of total soluble solids (TSsS) is pivotal in assessing the flavor quality of produce, serving as a critical marker of ripeness and quantifying the concentration of soluble sugars and minerals within the fresh produce, especially in tomatoes [55]. This study demonstrated that extended storage under ambient conditions significantly elevated the concentration of TSSs in tomatoes at a consistent rate (Figure 4B). However, the differences were not substantial. Likewise, there were minimal differences in TSS concentrations between samples coated with varying concentrations of CO. Sibomana et al. [56] observed an increase in the TSS level of tomatoes during extended storage at room temperature, and their results were correlated with reduced moisture content in the tomatoes. Munhuewyi [57] noted that the TSS increase in stored produce is primarily due to ripening and carbohydrate transformation, particularly under ambient conditions. This ripening leads to the breakdown of pectin into simpler sugars, thus elevating the TSS levels. In contrast, the TA level in the tomato samples was significantly decreased during extended storage at ambient conditions (Figure 4C). The control samples retained the lowest TA level as compared to other samples. The CS- and CS-CO-coated samples significantly retained the TA levels in the tomato samples. Similarly, the CS-CO-coated samples with varying concentrations also positively affected the retaining of the TA levels. Typically, the reduction in TA in plant crops stored under ambient conditions can be primarily attributed to metabolic reactions. This occurs as organic acids are utilized as substrates in the respiration and ripening processes alongside sugars [48]. The interaction between the TSSs and TA is vital for identifying the taste of the horticultural produce. Figure 4D shows the TSS/TA ratio changes of all tested tomato samples during storage. Overall, the TSS/TA ratio results for the tested samples showed an increasing trend throughout the storage period. Control samples had higher TSS/TA ratio values (*p* < 0.05) than the others. Meanwhile, CS- and CS-CO-treated samples exhibited no significant differences in their TSS/TA ratios during storage, with CS-CO-treated samples having slightly lower TSS/TA ratios. The variations in CO concentration did not significantly impact the TSS/TA ratio. The consistent TSS/TA values in the coated samples indicate that the coatings effectively moderated the storage-induced ripening in tomatoes.

### 3.5. Chlorophyll, Lycopene Content, and β-Carotene Content

**Figure 5:**
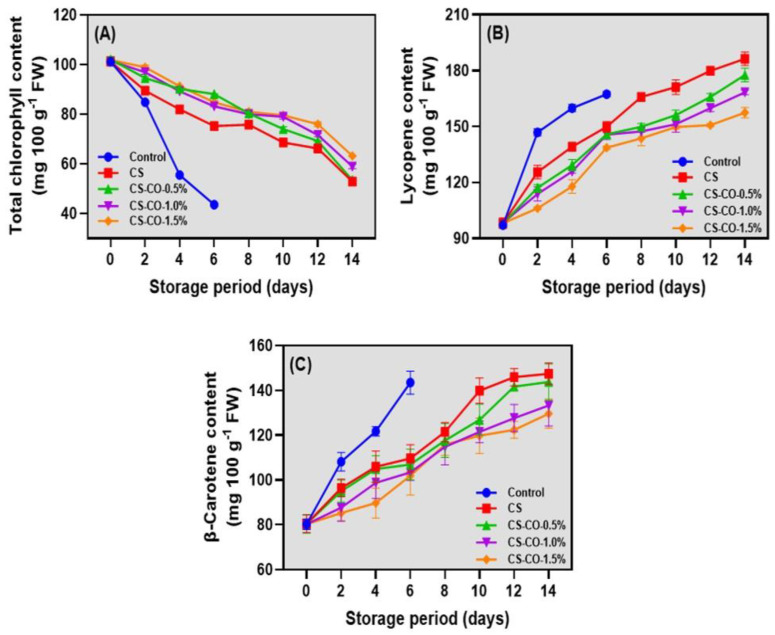
Changes in total chlorophyll (**A**), lycopene (**B**), and β-carotene (**C**) contents of the tomatoes treated with CS and CS−CO coating and stored under prolonged ambient conditions (~25 ± 1 °C). The results are presented as mean ± standard deviation (*n* = 3).

The changes in the chlorophyll, lycopene, and β-carotene content of the tomatoes coated with CS-CO at varying concentrations and stored under prolonged ambient conditions are shown in Figure 5. At the onset of storage, the chlorophyll content in the tomatoes was at a low level, suggesting they were at the breaker point where they transition from green to red [58]. Throughout the storage period, a consistent decline in chlorophyll levels was noted in the tomatoes, with the most pronounced reduction seen in the control samples, followed by the CS-coated samples (Figure 5A). Mandal et al. [59] reported that chlorophyll degradation and carotenoid synthesis lead to color changes in tomatoes during ripening due to the conversion of chloroplasts into chromoplasts. Two enzymes that regulate carotenoid production during ripening are the fruit-specific isoform phytoene synthase, which controls carotenogenesis, and D-xylulose 5-phosphate synthase, responsible for chlorophyll synthesis in green tissue and carotenoid synthesis in early ripening. Both enzymes exhibit increased gene expression during the ripening process. Furthermore, among the CS-based samples, the CS-CO samples had slightly better control over the degradation of chlorophyll contents than the CS-coated samples. However, at the end of the storage period, no differences in chlorophyll contents were found between all the CS- and CS-CO-coated samples. On the other hand, the lycopene and β-carotene levels in the tomato samples coated with CS-CO showed a continuous increase during storage (Figure 5B,C). An increase in the lycopene and carotenoid levels in tomatoes is considered an essential marker of maturity as they establish color changes [60]. Lycopene, a carotenoid hydrocarbon with the chemical formula C_40_H_56_, known for imparting on tomatoes their characteristic red color [61], demonstrated a steady increase in its levels throughout the storage period in the tomato samples. The results showed that the control samples exhibited higher lycopene and β-carotene levels than the treatments. This indicates the rapid changes in the maturation of the control samples at ambient storage. The lycopene content of the control and CS samples was higher than other samples (Figure 5B). On the other hand, the pigment changes in the tomatoes were significantly reduced during storage in the CS- and CS-CO-treated samples. Ronen et al. [62] highlighted the pivotal role of lycopene β-cyclase in the enzymatic conversion of lycopene into β-carotene, a process critical for determining the coloration of tomatoes. The present study found that tomatoes exhibited low levels of β-carotene during the initial storage phase, suggesting an inefficient conversion of lycopene and β-carotene. This observation was potentially due to the reduced activity of lycopene β -cyclase during this early storage period. CS-CO-treated samples exhibited lower β-carotene and lycopene values than the CS and control samples. Generally, the carotenoid contents in the tomato fruit, including lycopene and β-carotene, are significantly influenced by storage conditions such as temperature, atmosphere, and light, with controlled environments playing a pivotal role in their concentration [63]. The application of a CS-CO coating not only influences the environmental conditions surrounding the tomatoes but also regulates light permeation on the tomato surface, potentially controlling pigment changes [45].

### 3.6. AsA, TPC, and TAC

**Figure 6:**
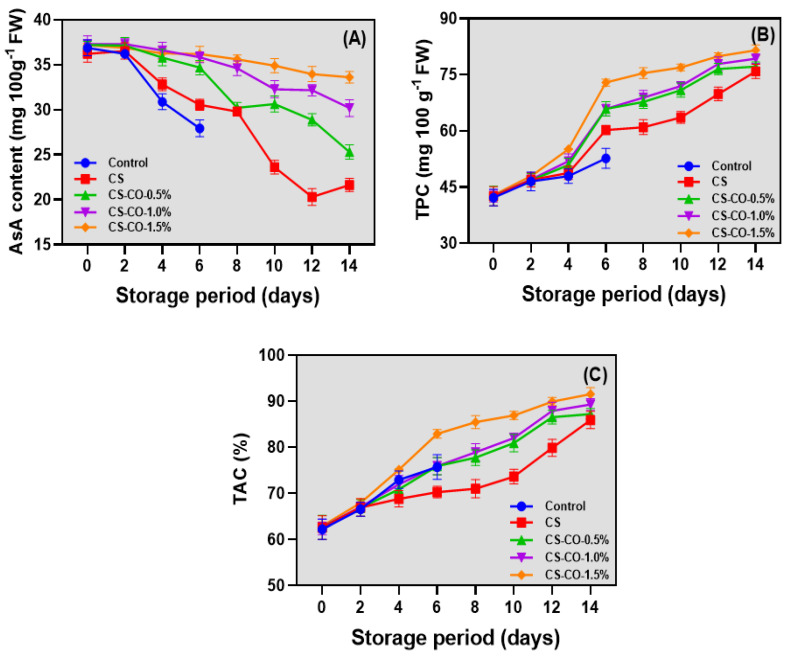
Changes in AsA (**A**), TPC (**B**), and TAC (**C**) of the tomatoes treated with CS and CS−CO coating and stored under prolonged ambient conditions (~25 ± 1 °C). The results are presented as mean ± standard deviation (*n* = 3).

The changes in the AsA level of tomatoes coated with CS-CO at varying concentrations and stored under ambient conditions are shown in Figure 6A. AsA is one of the predominant phytochemicals in tomatoes, and it increases in accordance with maturity levels [64]. At an optimum maturity level, AsA ranges between 20–40 mg per 100 g of fresh weight [65]. This is in accordance with the present study. Among the tested variables, there were not many differences in the AsA level at the beginning of storage; however, when the storage period progressed, the level of AsA was significantly decreased, and the loss was predominantly high in the control samples. Mandal et al. [59] observed a similar finding that the tomatoes significantly decrease their AsA levels in extended storage under ambient conditions. Compared with CS- and CS-CO-coated samples, the latter showed slightly better protection on the AsA level, and it was gradually higher as the CO concentration increased. Generally, tomatoes are susceptible to detrimental changes and loss due to temperature and oxidative stresses, and these stress conditions lead to the accumulation of reactive oxygen species (ROS) in the tomatoes [66]. AsA plays a pivotal role in mitigating this stress by acting as a scavenger, which helps alleviate the harmful effects of ROS. Consequently, the levels of AsA in the plants decrease as they get consumed in the process of neutralizing ROS [67]. In this study, tomatoes coated with CS-CO, particularly at higher CO concentrations, demonstrated improved control against AsA loss. However, the continuous decrease in AsA during storage might be linked to the senescence process of tomatoes. TPC are important secondary metabolites in tomatoes; next to AsA, TPC possesses crucial natural antioxidant properties and plays a significant role in offering a variety of health benefits [68,69]. The present study exhibited continuous increments in the TPC level in all the tested samples throughout the storage (Figure 6B). The TPC level was lower in the control and treated samples. Among the CS- and CS-CO-coated samples, a significant difference in TPC levels was noticed throughout the storage period. However, non-significant differences in TPC levels were observed between the CS-CO-coated samples, particularly those coated with CS-CO 0.5% and 1%. An increasing TPC level indicates an increased protective mechanism in the tomatoes as they need TPC to support pigmentation, reproduction, and growth, and it also exhibits resistance against microbial growth. Phenylalanine ammonia-lyase (PAL) is the critical enzyme that biosynthesises phenolics in plants, and it performs optimally in the presence of O_2_, and PAL catalyzes the phenylalanine to produce cinnamic acid, which is the precursor for the production of various phenolic compounds in the crops [70]. A low TPC level in the coated samples could form a barrier between the tomatoes and the environment and, thus, reduce the PAL enzymatic activities. Furthermore, this study found a continuous increase in the TAC level of the tested samples (Figure 6C). The CS-CO 1.5%-treated samples had the higher TAC level among the tested samples. Tomatoes treated with CS-CO at high concentrations exhibited a higher TAC activity. CS enhances food’s antioxidant activities and stability against oxidation due to its inherent properties and interaction with food components [71]. The higher TAC levels in CS-CO samples could also be due to the increased levels of AsA and TPC observed in these samples (refer to Figure 6A,B). AsA and TPC are well-known for their potent antioxidant activities. Additionally, the chemical components in CO, specifically eugenol, thymol, and cinnamaldehyde, demonstrate substantial antioxidant activities against DPPH radicals [72].

### 3.7. Microbial Growth

**Figure 7:**
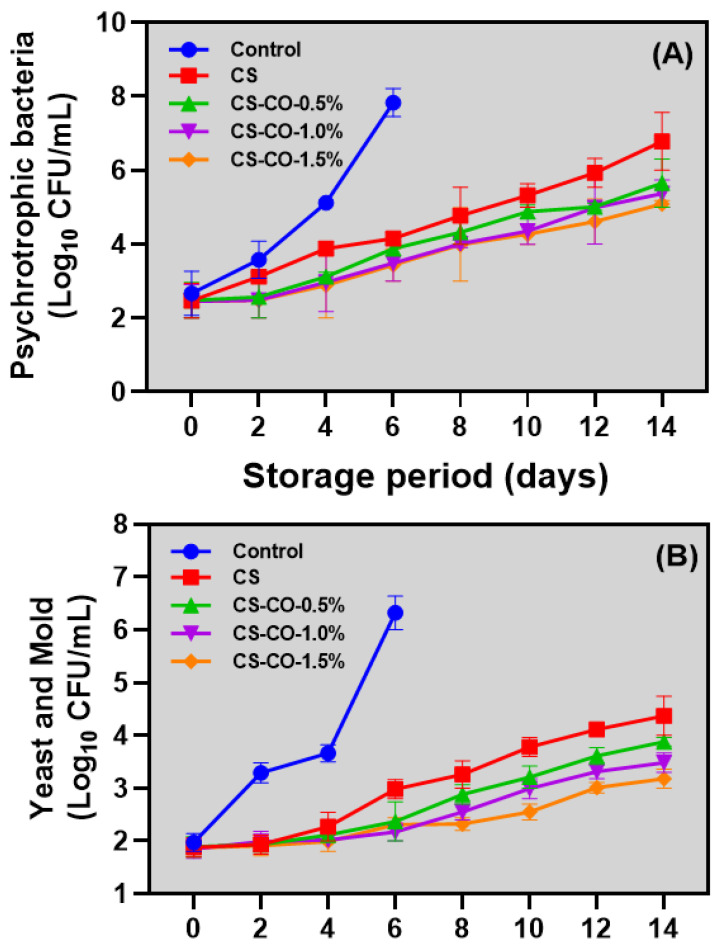
Changes in psychotropic bacteria (**A**) and yeast and mold (**B**) growth of the tomatoes treated with CS and CS−CO coating and stored under prolonged ambient conditions (~25 ± 1 °C). The results are presented as mean ± standard deviation (*n* = 3).

Tomatoes exhibit a relatively short shelf life in ambient conditions due to their substantial water and nutrient contents. These factors enhance physiological activities, increasing susceptibility to microbial attack and the predominant spoilage agents, including pathogenic fungi [73]. This study shows that the control samples could withstand a storage period of up to 6 days, after which storage was discontinued due to the exceedance of microbial growth on the fruit of more than 6 log10 CFU/mL. In contrast, the samples coated with CS and CS-CO at varying concentrations remained stable, and no visible microbial growth was observed until the end of the study period. Figure 7A represents the psychrotrophic microbial growth of the tomato samples, and, at 0 days of storage, the microbial growth of the tested tomatoes was between the range of 2.45 to 2.67 log10 CFU/mL (*p* > 0.05). The microbial counts of the control samples have been significantly higher since the second day of storage, and the growth continued to be high and reached an unacceptable level on the sixth day of storage (>7.8 log10 CFU/mL). On the other hand, CS-coated samples showed a lower level of microbial growth (6.78 log10 CFU/mL) at the end of storage (14 days), whereas the CS-CO samples significantly controlled the growth of psychrotrophic microbial counts compared to others and maintained the microbial growth levels below 5.65 log10 CFU/mL at the end of storage. Among the CS-CO samples, the suppression of psychrotrophic microbial growth was dose-dependent, with increased concentrations of CO showing significant control against psychrotrophic microbial growth. Similarly, the yeast and mold growth in the tomato samples coated with CO and CS-CO was significantly lower compared to the control samples (Figure 7B). At the beginning of the storage period, the yeast and mold growth in the samples ranged between 1.85 and 1.97 log10 CFU/mL. Among the variables tested, the control samples exhibited the highest growth, reaching an unacceptable level (>6.32 log10 CFU/mL) by the sixth day of storage. On the other hand, the CS- and CS-CO-coated samples were retained below 4.37 log10 CFU/mL values throughout the storage period.

The increased concentration of CO in the coating emulsion significantly controlled the growth of micro-organisms in the tomato samples. Studies have widely proven that the antimicrobial efficacy of CS and CO and their application to tomatoes could suppress the proliferation of micro-organisms, thereby lengthening the tomatoes’ shelf life at ambient conditions [74]. CS might induce ionic interactions in the microbial cell, induce the ionic imbalance and electrolyte leakage in the microbial cell membrane, and interfere with the synthesis of mRNA and protein in the micro-organism and, thereby, the death of micro-organisms [75]. Goy et al. [76] reported that CS effectiveness in microbial control is due to various mechanisms, such as cell wall disruption causing cell lysis, cytoplasmic membrane breakdown, electrostatic interactions between its positively charged glucosamine units and microbial cells’ negatively charged areas, and trace metal ion sequestration. Similarly, CO exhibits potent antimicrobial activity against various micro-organisms, mainly bacteria, yeast, and mold, and the potency of its inhibitory effect against microbial growth was found due to the abundance of cinnamaldehyde, eugenol, benzaldehyde, benzyl alcohol, benzoic acid, alpha phellandrene, linalool, vinyl acetate, and benzyl cinnamate [77]. Sarengaowa et al. [78] found that the addition of a higher concentration of CO significantly controlled bacterial growth. Vasconcelos et al. [79] found that CO and its compounds deter bacterial growth by altering lipid profiles, damaging cell membranes, disrupting ATPases, impeding cell division, affecting membrane porins and motility, and hindering biofilm development, alongside their anti-quorum sensing capabilities. Furthermore, Singla et al. [34] observed that using a CS-CO blend on pomegranate arils effectively suppresses aerobic micro-organisms.

## 4. Conclusions

The present study explored the efficacy of CS and CO coatings, particularly at varying concentrations of CO (ranging from 0 to 1.5%), in enhancing tomatoes’ shelf life and quality under ambient storage conditions. Integrating CO into CS-based edible coatings has significantly improved its properties and effectiveness. The CS-CO coatings, especially those with higher CO concentrations (1–1.5%), have been shown to preserve tomatoes’ quality, outperforming other tested variables in several key areas. Notably, the treated fruits exhibited excellent resistance to moisture loss, thus reducing the loss of fruit firmness compared to control fruits. Additionally, CS-CO coatings with higher concentrations (>1%) have effectively been modulating physicochemical qualities such as pH, TSS, TA, and the TSS/TA ratio, while also preserving essential phytochemicals including chlorophyll, lycopene, AsA, and TPC, thereby improving the TAC level in the treated tomatoes. The antimicrobial properties of the CS-CO coatings have also played a significant role in substantially inhibiting the growth of micro-organisms within the tomatoes. These findings underscore the potential of CS-CO coatings, particularly those with CO concentrations of 1–1.5%, as effective biomaterials for extending the post-harvest shelf life and enhancing the quality of the tomatoes, with broad implications for sustainable food preservation in the agricultural and food sectors.

## Data Availability

The original contributions presented in the study are included in the article, further inquiries can be directed to the corresponding author.
